# Supplementary material for: Breastfeeding outcomes in late preterm infants: A multi-centre prospective cohort study
Source: PLoS One. 2022 Aug 15;17(8):e0272583. doi: 10.1371/journal.pone.0272583 (PMC9377594; doi:10.1371/journal.pone.0272583)
Supplement: Attachment — Submitted filename: Response to Reviewers.docx [file pone.0272583.s001.docx]

*Reviewer #1:*

*1.Page 5, line 81 & 82: Indicate the specific recruitment date, completed date, and data collection finalised date*

All dates updated to exact date.

*2.Page 6, line 107: Is that any human milk or their mothers’ milk? Does this include donor breast milk?*

Methods clarified and wording updated;

Exclusive breastfeeding was defined as a baby receiving only breastmilk and medications, including oral rehydration solutions, vitamins and minerals, but no infant formula or non-human milk at each study time point and the week immediately preceding them.

*3.State the way you used to take consents for under 18 years old participants under ethical consideration section*

Ethical Considerations section updated;

Mothers of late preterm were approached in the Neonatal Units or Postnatal ward and informed consent was obtained for their infant/s. Ethics approval was granted by the Women’s and Children’s Health Network Human Research Ethics Committee HREC/18/WCHN/064. Site-specific and local governance approvals were obtained at each study site.

*4. Was the questionnaire validated in the local context and how?*

There wasn’t really one questionnaire for the study. Data at enrolment was obtained via maternal report in person, telephone or surveys that were designed as electronic data capture instruments. Short follow up surveys were sent to women at study timepoints (6 weeks, 3 months and 6 months) so that women could report on their breastfeeding practices remotely via text or email. Methods updated for clarity.

*5.Have you tested multicollinearity and the model fitness? If not reason? If yes, indicate it clearly.*

Relationships between measures of breastfeeding over time, or between exclusive breastfeeding and any breastfeeding at each time point were considered in separate statistical models. We have now explained in the methods that “There was no evidence of collinearity between characteristics or poor model fit in any of the multivariable models considered.” We have also clarified in the statistical methods that measures of breastfeeding (exclusive or any) were analysed in separate models at each time point (hence there were no concerns around collinearity in the breastfeeding outcomes over time).

*6. Table 1: what do you mean “born in Australia, (n=220)”? since your study was conducted completely @Australia.*

This is listed under maternal characteristics and refers to mothers born in Australia, table line headings have been updated for clarity.

*Reviewer #2:*

*Abstract - Avoid abbreviations in the abstract*

Abbreviations removed unless used multiple times, i.e. corrected age.

*Background -It would be better if you add the burden of the problem and the rationales why you are doing this research*

Further explanation added line 57 and 58: ‘Breastmilk is the optimal nutrition to support overall growth and development, gut maturation and immune protection. Complications relating to poor feeding are major contributors to the health burden in this population and one of the leading causes of extended hospital stay and readmission.’

*In the methods and materials section of the abstract- The type of model used to determine the factors associated with breastfeeding duration was not mentioned. I didn't understand why you used method and material? What materials did you use in this study?*

In the methods it states that “Associations between maternal and infant characteristics and breastfeeding at 6 weeks, 3 months and 6 months CA were assessed using multivariable logistic regression models (separate models for exclusive breastfeeding and any breastfeeding at each time point), with generalised estimating equations used to account for clustering due to multiple births. Effects are described as odds ratios (OR) with 95% CI. Maternal and infant characteristics were included in multivariable models based on factors associated with increased breastfeeding rates reported in the literature.”

Heading changed to Methods.

*The conclusion section of the abstract – I have confused by the findings stated in your result section and conclusion. Receipt of formula milk was associated with BF (breastfeeding) duration in the result section but in the conclusion part, early feeding practice and intention to breastfeed were predictors of BF duration?*

Sentence revised for clarity: Early feeding practices including the use of formula in hospitals, and intention to breastfeed >6 months were significant predictors of breastfeeding duration in this population.

*Outcome: line 104 - initial hospital length of stay and exclusive or any breastfeeding at 6 weeks, 3 months, and 6 months CA. what parameters do you use to categorize the outcome variable into 6 weeks,3 months, and six months?*

Key outcomes were exclusive or any breastfeeding at 6 weeks, 3 months, and 6 months. Surveys were sent to women at these timepoints. Initial length of stay was assessed but not in relation to feeding practices at 6 weeks, 3 months and 6 months. observation. This paragraph has been edited for clarity.

*- I couldn't find the adjusted odds ratio value in your multivariate logistic regression model to identify the factors associated with breastfeeding duration. if I was not mistaken, how could you conclude the factors associated with breastfeeding duration without making an adjusted odds ratio? you have made only bivariate logistic regression based on table 4.*

All odds ratios in Table 4 are from multivariable logistic regression models. For example, the odds ratios provided for centre are adjusted for duration planned to breastfeed, sex, NICU admission, formula as first milk feed and SEIFA. To make this clearer, we have now labelled the effect estimate as “Adjusted OR” in the table.

*Result –you have made too long and interrupted tables, and lacks description*

Tables re-formatted and description added.

*Table 3- breastfeeding at 6 months, (n=??? 248) - I am not clear about it? Generally, tables are not prepared in the way of attracting readers.*

Table 3 corrected and all tables modified for readability.

*Discussion*

*Line 212- Line 215 this paragraph lacks reference.*

This paragraph has been modified: Nevertheless, formula use during the neonatal admission may be modifiable with interventions such as pasteurised donor human milk or oral dextrose preparations. There remains little high-quality evidence to guide the clinical management of nutrition support in late preterm infants. Further research is warranted to understand the impact of adopting alternative strategies to maximise nutritional outcomes in this population.

*Line 218 – L 220. This statement lacks justification about exclusive breastfeeding at discharge related to an increase from 60 % to 74% and decreasing to 33% by 3 months of CA.*

This paragraph has been modified.

*Maternal intention to breastfeed>6 months is the most consistent predictor of breastfeeding duration. This factor was not well discussed with other research findings.*

Line 226 to line 234 in revised manuscript discusses intention to breastfeed: Maternal intention to breastfeed for >6 months was the most consistent factor associated with a longer duration of breastfeeding across all time periods. This is consistent with previous research, predominantly in term infants, and points to the need to promote breastfeeding antenatally to help women clarify their intentions. Our findings suggest there is room to improve support for women after they leave the hospital. Previous research has identified a lack of support from health care professionals in hospital and after discharge home and perceptions about low breastmilk supply as key barriers to breastfeeding late preterm infants. These barriers require addressing and are identified as a priority by parents and care providers.
