# Supplementary material for: Breastfeeding outcomes in late preterm infants: A multi-centre prospective cohort study
Source: PLoS One. 2022 Aug 15;17(8):e0272583. doi: 10.1371/journal.pone.0272583 (PMC9377594; doi:10.1371/journal.pone.0272583)
Supplement: Attachment — Submitted filename: Response_To_The_Reviewers.docx [file pone.0272583.s002.docx]

Thank you for your email received 7th April 2022 with additional comments from Reviewer 2. We appreciate the thorough review and feedback to improve our manuscript. Please find our responses below:

*Reviewer #2: The authors were not fully addressed my concerns especially in the abstract part of the manuscript. For instance,*

1. *In the methods and materials section of the abstract- The type of model used to determine the factors associated with breastfeeding duration was not mentioned. Subheading in the abstract is still method and material.*

A description of the model has been added to abstract methods: “Associations between maternal and infant characteristics and breastfeeding at 6 weeks, 3 months and 6 months CA were assessed using multivariable logistic regression models.”

Subheading updated to “methods”.

2. *The conclusion section of the abstract – I have confused by the findings stated in your result section and conclusion. Receipt of formula milk was associated with BF (breastfeeding) duration in the result section but in the conclusion part, early feeding practice and intention to breastfeed were predictors of BF duration?*

Abstract conclusion updated for clarity to: “Formula as the first milk feed and intention to breastfeed >6 months were significant predictors of breastfeeding duration.

*3. Table 1 and table 3 needs description in the frequency column*

Description added to frequency column in Table 1 and 3.
